# Implementing a Cross-Border Next-Generation Personal Health Record in the Philippines and Taiwan: An Implementation Case Report Using Health Level 7 International Fast Healthcare Interoperability Resources

**DOI:** 10.2196/56272

**Published:** 2025-07-02

**Authors:** Hsiu-An Lee, Jui-Chun Huang, Shih-Wun Huang, Wei-Han Chen, Alvin B Marcelo, Miguel Sandino O Aljibe, Chien-Yeh Hsu

**Affiliations:** 1 National Health Research Institutes, The National Institute of Cancer Research, Tainan, Taiwan; 2 Asia eHealth Information Network, Hong Kong, China (Hong Kong); 3 Standards and Interoperability Lab, Smart Healthcare Center of Excellence, Taipei, Taiwan; 4 Department of Information Management, National Taipei University of Nursing and Health Science, Taipei, Taiwan; 5 Medical Informatics Unit, University of the Philippines, Manila, Philippines; 6 Standards and Interoperability Lab, University of the Philippines, Manila, Philippines; 7 Department of Information Management, National Taipei University of Nursing and Health Science, 365 Mingde Road, Peitou District, Taipei, 112303, Taiwan, 886 939193212

**Keywords:** health insurance, personal health records, international patient summary, HL7 FHIR, cross-border healthcare, implementation study

## Abstract

**Background:**

Disparities in electronic health record systems hinder cross-border continuity of care, particularly where labor mobility and tourism intersect (eg, between the Philippines and Taiwan). Both nations collect claim data, yet neither fully aligns with international standards such as the Health Level 7 International, International Patient Summary (IPS).

**Objective:**

This implementation report aimed to convert health insurance data from Taiwan’s My Health Bank (MHB) and the Philippine Health Insurance Corporation’s Claim Form 4 (CF4) into a cross-border personal health record (PHR) aligned with the IPS using (Fast Healthcare Interoperability Resources) FHIR standards.

**Methods:**

We mapped each data element from CF4 (n=7 main sections) and MHB (n=12 major data items) to 17 sections of the IPS. We analyzed whether these elements matched IPS requirements (required or recommended or optional) and identified missing fields (eg, device use, social history, and advanced directives). We also designed a FHIR-based integration architecture, addressing system security with OAuth 2.0/SMART on FHIR and proposing a national uptake strategy for accelerating cross-border PHR implementation.

**Results:**

Of the 17 IPS sections, MHB covered 14 sections (82.4%), while CF4 covered 12 sections (70.6%). Both systems lacked sufficient data elements for medical devices, social history (eg, alcohol or tobacco), and advanced directives. We developed an implementation plan focusing on data interoperability, standardization, and privacy or security protocols. We propose a multiphase approach—beginning with the stakeholder engagement and pilot testing in both countries.

**Conclusions:**

Aligning CF4 and MHB data with IPS standards via FHIR can facilitate a robust cross-border next-generation PHR ecosystem. This approach may enhance patient safety, continuity of care, and policy development for the Philippines and Taiwan. Further collaboration, regulatory updates, and public awareness are vital to sustain integration and maximize patient benefits.

## Introduction

### Background

In recent decades, numerous eHealth technologies have become available, as countries have embarked on eHealth initiatives to promote health education and patient-centered care objectives [1]. The usage of personal health records (PHRs) has been associated with several benefits, including enhanced patient-provider relationships, increased patient engagement, improved medication adherence, positive health outcomes (such as better blood pressure and glycemic control), and greater organizational efficiency [2,3].

Under the aim of promoting patient safety, PHR facilitates the continuity of care [4]. When patients move to different countries or seek medical treatment abroad, having access to their health records ensures that health care providers can make informed decisions based on their medical history, allergies, medications, and previous treatments. In emergencies, such as accidents or sudden illnesses while traveling, having access to PHRs can be a matter of life and death. Medical professionals need immediate access to a patient’s health information to provide appropriate and timely treatment.

The evolution of PHRs into the next generation represents a significant shift in how individuals engage with and manage their health information. Unlike traditional PHRs, which often mimic digital versions of paper-based records, next-gen PHRs harness advanced technologies and data sources to offer a far more comprehensive and dynamic view of an individual’s health. Central to these next-gen PHRs is their emphasis on interoperability, designed to seamlessly integrate and share data with various health care providers, electronic health record (EHR) systems, wearable devices, and other health-related apps [5]. This interoperability ensures that health information is no longer confined within silos but can be readily accessed and shared with the authorized parties. Real-time data updates are a hallmark of next-gen PHRs. They provide continuous streams of health data, including vital signs, lab results, and medication adherence, often integrating with wearable devices and IoT (Internet of Things) sensors for ongoing monitoring [6,7]. Furthermore, these PHRs excel in data aggregation, pulling in health information from a multitude of sources such as EHRs, pharmacies, insurance claims, and even patient-generated data. This holistic view empowers users to track and manage their health more effectively. PHR should allow users to set health goals, track progress, and receive motivational feedback and reminders [8]. Within the broader health care ecosystem, next-generation PHRs often function as central hubs connecting patients with health care providers, pharmacies, laboratories, and insurers. Personalization is a key aspect. These personal health management tailor health recommendations and content to an individual’s specific health needs and goals [9]. In addition, users have greater control and ownership over their health data, deciding who can access their information and for what purposes. The next-generation personal health record is designed to empower individuals with greater control, access, and understanding of their health data while fostering improved communication and collaboration within the health care ecosystem. Through the integration of advanced technologies, it introduces a more dynamic and user-centric approach to personal health management.

The data sources for PHRs are diverse, including self-reports, long-term monitoring data, nutritional information, medical records, and more. Currently, common PHRs often separate into 2 kinds of data, self-reported or questionnaire data or clinical medical data. However, in practical applications, integrating this information holds great potential for providing more comprehensive and valuable medical information. In Taiwan, the Health Bank Book serves as a significant data source for PHRs, primarily deriving its content from health insurance reimbursement data [10]. These data are provided by validated sources and typically encompass crucial clinical diagnoses and medical service-related information. Therefore, the release of Health Bank Book data primarily follows an application framework focused on clinical diagnosis and medical services, providing robust reference information.

In recent years, there has been increasing collaboration across the Asia-Pacific region. From the perspectives of population and labor mobility, Taiwan has approximately 12 million workers, among which more than 700,000 are foreign laborers from Thailand, the Philippines, Indonesia, and Vietnam. Among them, around 150,000 are from the Philippines, primarily working in manufacturing (accounting for about 60% of foreign laborers) and domestic services or caregiving (approximately 36%).

Health insurance coverage in Taiwan is one of the labor rights granted to foreign workers, enabling effective cross-border exchange of medical data. This not only ensures the quality of medical care provided abroad but also safeguards the rights and well-being of workers from both sides.

According to the Philippines' Department of Tourism, between January and August 2024, approximately 155,086 Taiwanese tourists visited the Philippines, representing a 17.62% increase compared to the 131,849 visitors during the same period last year. Taiwan ranked sixth among the sources of international tourists to the Philippines, accounting for about 4.19% of the total number of international visitors.

In an increasingly globalized world, people frequently move across borders for work, education, and leisure. International health management ensures that individuals can access health care services and manage their health records seamlessly, regardless of their location. This is vital for expatriates, travelers, and immigrants. After the COVID-19 pandemic, changes in the epidemic have made us realize the importance of international cooperation. In the past, countries developed medical systems and provided technical support, knowledge exchange, and cooperative research with each other.

Studies show that design and adoption vary globally, with standardization efforts (including Health Level 7 International [HL7], Fast Healthcare Interoperability Resources [FHIR]) promising better interoperability [11]. From 2023 onward, new frameworks and policies (eg, 21st Century Cures Act in the United States) further encourage multinational data sharing [12].

However, future developments should move toward more comprehensive and integrated PHRs. This will facilitate the provision of diverse health information, enabling more forward-looking medical decisions while better meeting individual health needs. In this developmental process, we can draw from the experience of the Health Bank to ensure the accuracy and reliability of data sources for PHRs, providing enhanced medical and health management support for individuals.

### Objectives

The purpose of this study is to discuss the possibility of converting health insurance declaration data into PHRs and implement them in an internationally common format, and to explore the future adoption model of the international personal health management framework using health insurance data from Taiwan and the Philippines as examples.

## Methods

### Study Design

This implementation report followed a comparative design to evaluate whether Claim Form 4 (CF4; the Philippines) and My Health Bank (MHB; Taiwan) align with or can be adapted to the International Patient Summary (IPS). We systematically mapped all data elements from both forms to IPS’s 17 sections (required, recommended, and optional). We then proposed an architecture based on FHIR to enable cross-border interoperability. Finally, we formulated a multistakeholder national promotion strategy.

### Discover the Key Elements of the Next Generation of PHRs

According to changes in international cooperation and medical information architecture, PHRs are becoming more and more important. In addition to data content, there are many issues between system architectures that need to be paid attention to. Some key features and characteristics of next-generation PHRs are given in Textbox 1.

Textbox 1. **Key features and characteristics of next-generation personal health records (PHRs).**

- Interoperability: PHRs are designed to seamlessly integrate and share data with various health care providers, electronic health record (EHR) systems, wearables, and other health-related applications. This interoperability ensures that health information is not siloed and can be easily accessed and shared with authorized parties [13].
- Real-time data: Provide real-time updates of health data, including vital signs, lab results, and medication adherence. Integration with wearable devices and IoT (Internet of Things) sensors enables continuous data collection and monitoring [14].
- Data aggregation: These PHRs aggregate health data from multiple sources, such as EHRs, pharmacies, insurance claims, and patient-generated data. This comprehensive view allows users to track their health more effectively [15].
- Artificial intelligence (AI) and predictive analytics: Next-gen PHRs may leverage AI and machine learning algorithms to analyze health data. This can help users identify trends, receive personalized health recommendations, and even predict potential health issues [16].
- Security and privacy: Robust security measures are in place to protect sensitive health information. This includes encryption, 2-factor authentication, and compliance with health care privacy regulations like HIPAA (Health Insurance Portability and Accountability Act in the United States).
- Telehealth integration: Next-generation PHRs often include telehealth capabilities, allowing users to schedule virtual appointments with health care providers and access medical advice and consultations remotely [17].
- Health care ecosystem integration: These PHRs may serve as a central hub within a broader health care ecosystem, connecting patients with health care providers, pharmacies, laboratories, and insurers.

Overall, the next-generation personal health record aims to empower individuals with greater control, access, and understanding of their health data while facilitating better communication and collaboration within the health care ecosystem. It leverages advanced technologies to create a more dynamic and user-centric approach to personal health management.

### Implementation Framework

This study evaluates and inventories the feasibility of international data interoperability and explores collaborative measures for data application with the Philippines. The overall framework adopts a deductive approach, focusing on the data architecture. The selection process begins by identifying hospital-level or national-level datasets. The design prioritizes mapping essential fields to corresponding fields in international data formats, with all selections and analyses jointly inferred by the 2 research teams.

Our process emphasized (1) content mapping to IPS sections, (2) structural alignment with FHIR resources, (3) designing a security architecture using OAuth 2.0 or SMART on FHIR, and (4) drafting a national adoption strategy. We consulted HL7’s official IPS Implementation Guide [11] and relevant FHIR documentation for best practices.

### International PHR Adoption

In order to achieve international data exchange and health record sharing, the adoption of internationally open standards and data content will accelerate development. This approach allows different systems and countries to have a common architecture for implementation, and reduces differential communication and comparison between different suppliers.

The adoption of FHIR has gained significant momentum in the health care industry due to several key factors, including interoperability, simplicity, modularity, community support, government mandates, and scalability. FHIR was developed to address the interoperability challenges in health care. Its standardized approach to data exchange allows health care systems and applications to communicate and share data seamlessly. This is crucial for improving patient care, reducing medical errors, and enhancing the overall efficiency of health care processes.

The straightforward and modern RESTful application programming interface approach was used to implement it. Its resource-based model is based on common web standards like HTTP, JSON, and XML, making it accessible to a wide range of developers and organizations. All structures are designed with a modular approach, where health care data is organized into discrete resources (eg, patients, medications, and observations). This modular approach enables the gradual adoption of FHIR resources based on specific needs, making it flexible and adaptable for different health care environments. The fundamental nature of FHIR allows it to accommodate both small clinics and large health care systems. This makes it an attractive choice for a wide range of health care providers.

Since there is a large group of volunteers and international organizations supporting it, FHIR is backed by a strong and active international community. Mainly developed and maintained by HL7, a global authority in health care standards. The collaborative nature of FHIR development ensures that it remains up-to-date and relevant. In terms of policy, many countries have recognized the importance of health care interoperability and have implemented regulations that encourage or mandate the use of FHIR. For example, the United States has included FHIR as part of the interoperability rules through the 21st Century Cures Act.

An IPS will be adopted in this study. The IPS is a standardized and structured health summary document designed to improve the exchange of essential patient information across borders and different health care systems. It aims to ensure that vital patient data is accessible to health care providers, particularly in emergency situations or when patients are seeking medical care in foreign countries. An IPS document serves as an EHRs extract containing crucial health care information about an individual’s care. It adheres to the standards outlined in EN 17269 and ISO 27269 [18], primarily designed to support scenarios involving “unplanned, cross-border care,” while its utility extends beyond this scope. The IPS document is crafted to be globally applicable, transcending regional or national boundaries. The IPS dataset is intentionally concise and not exhaustive, making it suitable for any medical specialty and independent of specific health conditions while remaining clinically pertinent.

IPS follows a standardized format and structure, making it easy for health care providers to find and understand critical patient information quickly. The essential health information such as allergies, current medications, existing medical conditions, recent procedures, immunization history, and contact details is included. The whole document is designed to be multilingual, ensuring that health care providers from different linguistic backgrounds can access and comprehend the patient’s health information. IPS is particularly valuable in emergency situations where patients may not be able to provide their complete medical history, allowing health care providers to make informed decisions about their care.

For data exchange and transformation, IPS should can be integrated into various health information systems and EHRs. The secure and privacy compliant should be included into system which designed for IPS. IPS adheres to privacy and security standards, ensuring that patient data remains confidential and protected during exchange.

The IPS is part of broader efforts to improve health information exchange at the international level, promoting better patient care and safety, especially for individuals who receive medical treatment in foreign countries or travel extensively. It contributes to the standardization of health data formats and facilitates more seamless health care delivery across borders.

### PHR Data Source

#### Taiwan MHB

Taiwan MHB is a government-initiated online platform in Taiwan that serves as a personal health record system for citizens. It is designed to provide individuals with easy access a wide range of their health-related data which includes diagnosis, medication, treatment from outpatient, clinic, dental, traditional Chinese medicine, allergy information, inspection (check) result, imaging or pathological examination (examination) report information, summary of discharge medical records, organ donation or palliative care wishes, adult preventive, vaccination, and 4 cancer screening results. All the data is collected from various health care sources for health insurance claims which include hospitals, clinics, pharmacies, and laboratories. The platform aggregates health data from multiple health care providers and sources, providing users with a centralized and holistic view of their health history. This integration helps individuals and healthcare professionals make informed decisions about their health care.

Users can view their prescription history and medication information, helping them keep track of their prescribed drugs and potential drug interactions. Taiwan MHB offers health promotion and education materials to help users make informed decisions about their health and well-being. It provides information on various health topics and encourages preventive health care measures.

An individual’s National Health Insurance card and a personal identification number can be used to secure access Taiwan MHB. This ensures that only authorized users can access their health records. It also provides mobile applications to make it convenient for users to check their health information on-the-go.

#### Philippine Health Insurance Corporation

Established in 1995, the Philippine Health Insurance Corporation (PhilHealth) plays a pivotal role in realizing universal health coverage within the Philippines. As a tax-exempt, government-owned and controlled corporation attached to the Department of Health, PhilHealth is the primary governmental agency entrusted with the administration of the nation’s health insurance program. Its fundamental mission is to furnish health insurance coverage and benefits to the Filipino populace, guaranteeing access to vital medical services and financial security against the often-burdensome expenses associated with healthcare.

The purview of PhilHealth encompasses a broad spectrum of individuals, including employees, self-employed persons, and sponsored members such as indigent families and senior citizens. Its comprehensive coverage extends to a diverse array of health care services, encompassing hospitalization, outpatient care, preventive health measures, and maternity benefits, among other critical provisions. The overarching goal is to enhance accessibility to health care, particularly for those in vulnerable circumstances, achieved through strategic partnerships with various health care providers, including both public and private institutions.

Recognizing the need for equitable health care access, PhilHealth extends its coverage to different categories of beneficiaries, ensuring inclusivity across society. The “Formal” sector encompasses individuals employed by public and private entities, while the “Indigents,” also known as “PhilHealth Ng Masa,” benefit from national government subsidies via the National Household Targeting System for Poverty Reduction. “Sponsored Members” receive support from their respective Local Governments, while “Lifetime” members, comprising retirees and pensioners with at least 120 months of premium payments, are accorded nonpaying status.

In addition, the “Senior Citizen” category, as stipulated under RA 10645, offers free PhilHealth coverage to all Filipino citizens aged 60 and above. Meanwhile, the “Informal Economy” classification accommodates informal sectors, self-earning individuals, organized groups, Filipinos with dual citizenship, natural-born citizens, and even overseas Filipino workers within its fold.

PhilHealth’s enduring commitment to fostering equitable access to health care services continues to drive its initiatives, striving to safeguard the health and well-being of the Filipino population at large.

PhilHealth CF4 is used by organizations to apply for health insurance reimbursement, and it contains 7 main sections. Since PhilHealth has not yet provided people with PHRs, CF4 data will be used in this study to evaluate whether it is suitable to develop a PHR framework for public use and compare it with IPS.

### Ethical Considerations

This study did not involve any human subject research. The system development and scenario testing were conducted using simulated data solely generated for this research purpose. Therefore, the study did not require institutional review board approval. The data used in this study were fully synthetic and created by the research team. As such, there were no issues related to informed consent, data authorization, or secondary data usage. Since the dataset was entirely simulated and did not include any real individual or patient information, there were no privacy or confidentiality concerns associated with the research. No human participants were involved in this study; hence, there was no compensation provided. Finally, the study did not include any identifiable images or visual materials derived from real individuals. All figures and visual content are based on simulated or generic representations, and there is no risk of identification.

In ethics review or approval, the data sets examined (CF4 and MHB) involve health insurance claim information. No identifiable patient-level data were directly used in this implementation study. Because only de-identified, aggregated, and publicly or institutionally authorized data were analyzed, the study qualified for exemption per local institutional review board guidelines. In informed consent was not required from individual patients as data were anonymized and retrospective.

In context with privacy and confidentiality, no patient names, addresses, or unique identifiers are presented or shared. The study team adhered to data protection protocols and removed any potentially identifiable information before analysis. Compensation was not provided, as no direct participant enrollment was required. This manuscript does not include identifiable patient images.

## Results

### Overview

In this study, the FHIR international standard format is used to implement the content of IPS.

This study investigates the development of the medical information industry in the United States, Singapore, Japan, and Indonesia. Many manufacturers have developed electronic medical record systems that use FHIR as the data exchange standard, as described in Table 1.

**Table 1.** Major health information standards and prominent health IT vendors by country.

Country	Standard	Manufacturer
Unite States	HL7,^a^ FHIR^b^	Epic Systems, Cerner, Allscripts Healthcare Solutions, Athenahealth, McKesson
Singapore	HL7, DICOM. IHE	Integrated Health Information Systems, Philips Healthcare, InterSystems, NCS Group
Japan	HL7, DICOM, JAMI	HITACHI, Fujitsu, NEC, NTT DATA, Mitsubishi Electric
Indonesia	HL7, DICOM. IHE	Jireh Group, BMSC, HSC Medical Center, MAKNA Cancer Research Institute

a HL7: Health Level 7 International.

b FHIR: Fast Healthcare Interoperability Resources.

c DICOM: Digital Imaging and Communications in Medicine

d IHE: Integrating the Healthcare Enterprise

Among them, Epic Systems and Cerner in the United States have outstanding achievements. In the Newsweek world best smart hospital 2023 survey, EPIC’s system was used in 6 out of the 10 top-ranking hospitals, and it was used in 14 out of the amount top 20 hospitals.

Oracle Cerner leads the EHR global market according to the 2021‐2022 Global Growth Report presented by Cerner. The KLAS 2022 global (non-US) electronic medical record market report states that Oracle Cerner’s customers include 2389 acute inpatient hospitals, of which 1052 are non-US customers, including medical institutions in the Middle East, the United Kingdom, Canada, and Australia.

### FHIR International Standard Adoption

FHIR is adopted to enhance interoperability among health care systems, enabling different health care providers, systems, and applications to seamlessly exchange and share patient data. This interoperability is crucial for delivering better patient care, reducing errors, and improving efficiency. It offers a standardized way to represent and exchange health care information. Through the standardization simplifies data integration and reduces the complexity of data exchange, making it easier for health care organizations to work together. Through the FHIR, patient-centric care can be easier reached. This leads to more informed decision-making and better patient outcomes. For the technology and system development part, the uses modern web standards which include JSON and RESTful application programming interfaces, making it compatible with today’s technology stack. This compatibility encourages its adoption as it aligns with current IT trends. Furthermore, many regulations discuss the application of FHIR, which all require data exchange and operational protection to be completed, such as the Health Insurance Portability and Accountability Act (HIPAA) and the 21st Century Cures Act in the United States.

There are many international companies across the healthcare spectrum that have integrated FHIR into their systems to enhance interoperability and patient care.

Epic Systems, Cerner, and Allscripts have embraced FHIR to improve data exchange in EHRs systems, And health insurance companies like Aetna and UnitedHealthcare use FHIR to enable secure data exchange with health care providers. In Europe, Orion Health and InterSystems are implementing FHIR to promote cross-border data exchange and interoperability.

### Data Content of IPS FHIR

**Figure 1.**
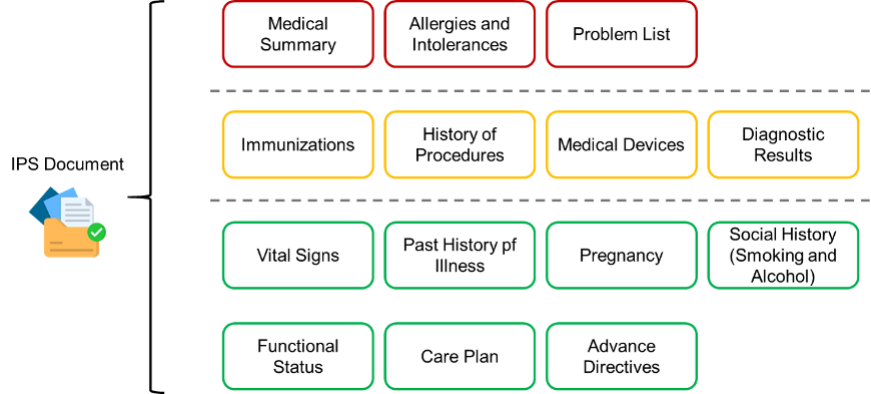
The structure of the International Patient Summary (IPS).

A team has already defined the FHIR IPS implementation guild based on the IPS specifications. This implementation guide is used to compare and use data fields and select necessary items. Include necessary items such as medications, allergies, and disease status; it is recommended to include information such as vaccinations, treatment history, medical equipment, diagnostic reports, etc; additional options include vital signs, disease history, pregnancy status, smoking and alcohol habits, care plan, functional status, and advance directives (Figure 1).

### Data Mapping Outcomes

IPS defines 17 sections (5 required, 7 recommended, and 5 optional). Table 2 details how CF4 and MHB compare. In summary, MHB fully or partially covered 82.4% (14/17) IPS sections. Missing elements primarily related to device usage, social history (tobacco and alcohol), functional status, and advance directives.

CF4 covered 12/17 (70.6%), with additional limitations regarding unstructured text fields, past history of illnesses, radiology or pathology results, and social history.

Consequently, substantial modifications or additional data capture would be needed to meet all IPS-required or recommended sections.

**Table 2.** Comparison of IPS, CF4, and MHB data content and architecture (This table compares how each data element in IPS aligns—or does not align—with CF4 and MHB. It includes required, recommended, and optional sections. [O=presentor easily mapped, X=absentor limited.]).

Section	Type	IPS^a^	PhilHealth CF4^b^	Taiwan MHB^c^
Header	Required	Patient	O	O
Header	Required	Encounter	O	O
Medication summary				
	Required	Medication request	O	O
	Required	Medication statement	O	O
	Required	Medication	O	O
Allergies and intolerances	Required	Allergy intolerance	O	O
Problem list	Required	Condition	O	O
Immunizations	Recommended	Immunization	O	O
History of procedures				
	Recommended	Procedure	O	O
	Recommended	Organization	O	O
	Recommended	Device	X	X
Medical devices				
	Recommended	Device use statement	X	X
	Recommended	Device	X	X
Diagnostic results				
	Recommended	Observation (results)	O	O
	Recommended	Diagnostic report	O	O
	Recommended	Organization	O	O
Laboratory results				
	Optional	Observation results: laboratory	O	O
	Optional	Specimen	X	X
	Optional	Media observation (results: laboratory and media)	X	O
Radiology results				
	Optional	Observation (results: radiology)	X	O
	Optional	Device	X	X
	Optional	Imaging study	X	O
	Optional	Practitioner	X	O
Pathology results				
	Optional	Observation results: pathology	X	O
	Optional	Specimen	X	X
	Optional	Media observation	X	O
Vital signs	Optional	Observation	O	O
Past history of illnesses	Optional	Condition	X	O
Pregnancy				
	Optional	Observation (pregnancy: EDD)	O	O
	Optional	Observation (pregnancy: outcome)	O	O
	Optional	Observation (pregnancy: status)	O	O
Social history (SH)				
	Optional	Observation (SH: tobacco use)	X	X
	Optional	Observation (SH: alcohol use)	X	X
Functional status (autonomy or invalidity)				
	Optional	Condition	X	X
	Optional	Clinical impression	X	X
Plan of care	Optional	Care plan	X	X
Advance directives	Optional	Consent	X	X

a IPS: International Patient Summary.

b CF4: Claim Form 4.

c MHB: My Health Bank.

d EDD: Estimated date of delivery.

### Security and Architecture Implementation

We adopted OAuth 2.0 as the primary authorization mechanism, with SMART on FHIR providing user-level permissions and token-based access to patient data. This approach ensures robust privacy controls consistent with HIPAA-aligned protocols. Both CF4 and MHB would require re-engineering to issue FHIR resources, including (1) FHIR resource mapping, that is patient resource aligns well with CF4’s identification data and MHB’s beneficiary info; (2) medication and allergies from MHB integrate readily with FHIR’s medication statement or allergy intolerance resources, that is, CF4 has partial coverage requiring structured fields; and (3) cross-border data flow, that is, a pilot exchange between Taiwanese and Philippine health care providers could test read or write operations on a shared FHIR server.

### PHR Management Structure

To accelerate the development of personal health record management globally and facilitate international data integration, we propose adopting a personal health record content based on the IPS and using FHIR as the international standard. Throughout the development process in various regions, we have also compiled recommendations from WHO (World Health Organization) guidelines and international organizations, emphasizing the following key areas mentioned in Textbox 2.

Textbox 2. **Key areas.**

- Standardization: Establishing standardized formats and protocols for health data to ensure interoperability across different systems and countries.
- Data security and privacy: Implementing robust security measures and privacy safeguards to protect sensitive health information.
- Interoperability: Ensuring seamless integration with various health information systems, both at the national and international levels.
- Inclusion of key health information: Incorporating essential patient information, medical history, medications, allergies, immunizations, and relevant diagnostic results.
- Patient access and control: Enabling patients to access and control their health records, allowing them to share information securely with health care providers.
- Cross-border health data exchange: Facilitating the exchange of health information across borders to support continuity of care for individuals who travel or receive medical services internationally.
- Integration with existing health systems: Ensuring compatibility and integration with existing EHR systems, health information exchanges, and other healthcare infrastructure.
- Technological standards: Adhering to international standards for health informatics to enhance consistency and compatibility.

### National Participation Process

In the promotion of national strategies, there are various facets to consider. Drawing insights from cases in the United States, Europe, and international corporations, key implementation points for national initiatives can be summarized as follows:

In the planning and development process, it is imperative to engage various stakeholders such as health care providers, government agencies, insurers, and patient advocacy groups. These entities should be invited to join the promotion group, contributing their expertise to provide critical solutions for the market. Regulatory compliance is essential, involving alignment with national and international regulations and standards related to health data management and interoperability. Furthermore, a comprehensive approach includes public awareness and education through campaigns that inform the public about the benefits and implications of the comprehensive international PHR.

User feedback and input play a crucial role, with efforts focused on soliciting opinions from end-users, including patients and health care professionals, to ensure the system aligns with their needs and expectations. Practical testing is facilitated through the implementation of pilot programs, evaluating the functionality, security, and usability of the comprehensive international PHR in real-world settings. Finally, policy development is integral, requiring the formulation of policies and guidelines governing the use, access, and sharing of international health records to establish a robust legal framework. This holistic approach ensures the involvement of key stakeholders, regulatory compliance, public awareness, user input, practical testing, and the establishment of necessary policies for the successful development and implementation of the comprehensive international PHR.

## Discussion

### Principal Findings

This research focuses on advancing the development of an international PHR by analyzing health insurance claims records. The study investigates the differences between the health insurance claim form used by PhilHealth in the Philippines and the MHB in Taiwan. By comparing these 2 schemas to the IPS, the research is able to identify gaps and overlaps that are important when transforming CF4 and MHB into a comprehensive PHR.

Using a comparative analysis, the study identifies key data elements present in international patient summaries that are lacking or underrepresented in the current systems. The aim is to optimize the effectiveness of CF4 and MHB by incorporating essential messages that align them with the functionalities of a PHR. The findings reveal gaps in converting health insurance claims into PHRs, emphasizing the importance of addressing missing components for holistic care, patient safety, and continuity of care.

### Comparison to Previous Work

In terms of data fields, Taiwan’s MHB has undergone years of adjustments and now possesses a comprehensive structured framework that can be effectively converted into the FHIR IPS format. In contrast, the data structure of the Philippines’ CF4 is relatively less developed, with many fields stored in unstructured text formats, leading to significant limitations in data usability.

Each country should conduct a thorough inventory of its medical data to ensure maximum consistency and redundancy. This approach would give greater significance to data exchange and usage.

For addressing international medical needs, it is essential to conduct an in-depth assessment of data usability and standardization. By converting data into international formats, greater interoperability can be achieved [19,20].

### Strengths and Limitations

Strengths include a direct comparison of 2 national frameworks (CF4 and MHB) to internationally recognized standards. Limitations involve the largely conceptual nature of the cross-border PHR architecture and the reliance on partial unstructured data in CF4. Further pilot testing and real-world implementation remain future work.

### Future Directions

This study has confirmed that the IPS is a sufficient reference standard for health data exchange in international applications. The next phase should involve engaging with more countries to discuss feasible data sources, standardized data preprocessing workflows, details of textual data extraction, and reliable methods for data exchange. Furthermore, more advanced discussions should focus on data integration and consistency standards.

### Conclusion

By mapping CF4 and MHB to IPS sections, this implementation study highlights the feasibility of developing a shared FHIR-based next-generation PHR for the Philippines and Taiwan. Such an approach can improve health care coordination, especially for foreign workers and tourists, through seamless exchange of medical data. Adoption hinges on robust policy, stakeholder collaboration, and structured data collection to fill existing gaps in coverage.

## Supplementary material

10.2196/56272 Checklist 1 iCHECK-DH: Guidelines and Checklist for the Reporting on Digital Health Implementations.

## Abbreviations

CF4: Claim Form 4
EHR: electronic health record
FHIR: Fast Healthcare Interoperability Resources
HIPAA: Health Insurance Portability and Accountability Act
HL7: Health Level 7 International
IPS: International Patient Summary
MHB: My Health Bank
PHR: personal health record
WHO: World Health Organization
